# Influence of Dynamic Strength Index on Countermovement Jump Force-, Power-, Velocity-, and Displacement-Time Curves

**DOI:** 10.3390/sports5040072

**Published:** 2017-09-23

**Authors:** John J. McMahon, Paul A. Jones, Thomas Dos’Santos, Paul Comfort

**Affiliations:** Directorate of Sport, Exercise and Physiotherapy, University of Salford, Frederick Road, Salford M6 6PU, UK; p.a.jones@salford.ac.uk (P.A.J.); t.dossantos@hotmail.co.uk (T.D.S.); p.comfort@salford.ac.uk (P.C.)

**Keywords:** ballistic training, strength training, temporal phase analysis, athlete monitoring

## Abstract

The dynamic strength index (DSI), often calculated as the ratio of countermovement jump (CMJ) propulsion peak force to isometric mid-thigh pull (IMTP) peak force, is said to inform whether ballistic or maximal strength training is warranted for a given athlete. CMJ propulsion peak force is highly influenced by jump strategy, however, which is not highlighted by the DSI alone. This study aimed to quantitatively compare CMJ force-, power-, velocity-, and displacement-time curves between athletes who achieved high versus low DSI scores. Fifty-three male collegiate athletes performed three CMJs and IMTPs on a force platform. Athletes were ranked based on DSI score and the CMJ kinetic and kinematic-time curves of the bottom and top twenty athletes were compared. The low DSI group (0.55 ± 0.10 vs. 0.92 ± 0.11) produced greater IMTP peak force (46.7 ± 15.0 vs. 31.1 ± 6.6 N·kg^−1^) but a larger braking net impulse in the CMJ, leading to greater braking velocity and larger countermovement displacement. This strategy resulted in a similar CMJ propulsion peak force (25.9 ± 2.2 vs. 25.4 ± 3.1 N·kg^−1^) to the high DSI group. These results, taken together with those of previous studies, support the notion of ballistic versus maximal strength training likely being better suited to low versus high DSI scorers, respectively.

## 1. Introduction 

To provide insight into an athlete’s training status, and thus inform future training focus, the ratio of propulsion peak force produced during ballistic vertical jumping (either the squat jump (SJ) or countermovement jump (CMJ)) to isometric peak force produced during the isometric mid-thigh pull (IMTP) has been recommended in the literature [1,2,3,4,5]. The resultant ratio, which is termed the dynamic strength index (DSI) or dynamic strength deficit, typically yields high reliability (intraclass correlation coefficient (ICC) of ≥0.92) and low variability (coefficient of variation (CV) of ≤4.6%) [2,5,6] and is said to inform whether ballistic or maximal force development is warranted for a given athlete. As isometric peak force capacity is higher than propulsion peak force capacity (due to the force-velocity characteristic of muscle), a DSI of ≤0.60 is suggested to indicate that ballistic training is warranted as only 60% of the athlete’s maximal isometric force capacity is being utilized during a ballistic jump, whereas a ratio of ≥0.80 indicates that maximal strength training is warranted as the athlete is utilizing ≥80% of their full isometric force capacity during a ballistic jump (so the most effective strategy would be to increase isometric force capacity) [2]. Of course, relative isometric force capacity (i.e., relative strength) must also be considered alongside DSI values to better inform future training priorities for a given athlete, as one could produce a DSI of ≤0.60 but also be very weak, thus maximal strength rather than ballistic (or concurrent) training may be more suitable [7]. 

The DSI seemingly represents quite a simplistic approach to athlete ‘strength diagnostics’ and it is currently unknown whether this is efficacious. If an athlete produces a maximal effort during the IMTP then the peak force produced should accurately reflect how much force said athlete can voluntarily produce isometrically with their legs. Propulsion peak force produced in the SJ or CMJ is largely influenced by jump strategy, however, not just strength [8]. For example, adopting a compliant leg strategy in the CMJ by increasing countermovement displacement or starting the SJ from a deeper squat position acts to decrease propulsion peak force, but can increase jump height through the application of a net impulse that is characterized by a longer time of force application [9,10]. Therefore, the DSI does not reveal how much an athlete could utilize their force capacity in the SJ or CMJ, but rather how they expressed force in the testing that they participated in. Indeed, applying a propulsion net impulse characterized by a smaller force applied over a longer time would likely not be useful for athletes whose sporting actions are typically time constrained (i.e., they are required to produce large forces over short times). It is important to note that the specific instructions given to athletes for the CMJ will influence this, with emphasis on jump height alone typically leading to the aforementioned short (low force) and fat (long time) style of impulse generation whereas emphasizing fast movement (short movement times) whilst still aiming for maximal jump height usually results in a larger force and a shorter time to take-off [11]. Nevertheless, a preferred CMJ strategy was recently shown to yield better DSI values compared to those attained for the SJ [5], illustrating that athletes tend to demonstrate a consistent CMJ technique. 

The fact that propulsion peak force produced in vertical jumps can be influenced by countermovement amplitude (CMJ) or starting squat depth (SJ) [9,10] and that propulsion peak force represents just one instantaneous ‘gross’ value of force produced throughout the entire propulsion phase [12] presents a major limitation of the DSI calculation. Although it is easier to standardize starting squat depth in the SJ, starting knee joint angles have varied from 45° to 110° in previous studies [2,5,6] and it is also often difficult to prevent small amplitude countermovements from occurring prior to the propulsion phase in this jump [13]. This may be why DSI values derived from a CMJ were shown to be more reliable in a recent study [5]. The athletes’ preferred countermovement amplitude (i.e., depth) and their intention to jump as fast and as high as possible has been enforced in studies which have calculated the DSI using the CMJ [3,4,5,14,15]. Nevertheless, preferred countermovement amplitude differs widely across athletes [11,16,17] and can alter due to undertaking different training regimens [7,18,19]. It is likely important, therefore, to consider the preferred jump strategy (i.e., propulsion displacement/velocity etc.) adopted by athletes alongside their associated propulsion peak force values when interpreting any resultant DSI scores. This could be achieved by completing a temporal phase analysis (TPA) which enables a quantitative description of how force-, power-, velocity-, and displacement-time curves differ throughout the entire jump with respect to changes (same athlete) or differences (between athletes) in DSI scores [11,12,20].

A TPA approach would lend insight into how propulsion peak force was achieved in the vertical jump (assuming peak force was genuinely achieved in the IMTP) by those with a high or low DSI score. This will inform the general strategy performed by each of those groups and question the assumption of the DSI ratio in terms of whether low and high DSI scores reflect a jump strategy that would likely benefit most from ballistic and maximal strength training, respectively, based on what we ‘know’ to be reflective of a desirable jump strategy (i.e., an impulse characterized by a high force and short time). The primary purpose of this study was, therefore, to quantitatively describe the influence of DSI on CMJ (given it yields better DSI reliability than the SJ [5]) force-, power-, velocity‑, and displacement-time curves by comparing these curves, using the TPA approach, between athletes who achieved differing (i.e., high versus low) DSI values. It was hypothesized that a high DSI would be associated with larger force and power but lower velocity and countermovement displacement both in terms of the peak values attained and throughout large portions of the unweighting, braking, and propulsion phases of the CMJ.

## 2. Materials and Methods

### 2.1. Subjects

Fifty-three male collegiate athletes (who competed primarily in soccer or rugby union) were recruited to participate in this study. Each subject attended a single testing session (cross-sectional study design) in a laboratory setting at approximately the same time of day. All subjects gave their informed consent for inclusion before they participated in the study. The study was conducted in accordance with the Declaration of Helsinki, and the protocol was approved by the Institutional Ethics Committee (HSCR16/36). Subjects were ranked based on DSI scores and then split into high (top 20 subjects) and low (bottom 20 subjects) DSI groups post-testing. Dividing the subjects in this manner resulted in the high and low DSI groups’ mean DSI scores being equal to one standard deviation above and below, respectively, the mean DSI score attained by all subjects tested (*n* = 53). This method of splitting groups was utilized in a recent study conducted in our lab [11]. The physical characteristics and resistance training experience of all subjects and those placed in each group can be seen in Table 1.

### 2.2. Procedures

Following a brief warm-up (~10 min) consisting of dynamic stretching and sub-maximal jumping, subjects performed three CMJs (interspersed with one minute of rest) to a self-selected depth. Subjects were instructed to perform the CMJ as fast and as high as possible, whilst keeping their arms akimbo. Any CMJs that were inadvertently performed with the inclusion of arm swing or leg tucking during the flight phase were omitted and additional CMJs were performed after one minute of rest.

For the IMTPs, subjects adopted a posture that replicated the position at which they would start the second pull phase of the clean, with their knee and hip angles within 140–150°, in line with previous research [21,22,23]. An immovable, collarless cold rolled steel bar was integrated with a portable IMTP rig and positioned at mid-thigh level (Fitness Technology, Adelaide, Australia). Once the bar position was established, the subjects stood on the force platform, and their hands were strapped to the bar using standard lifting straps. Each subject then performed two warm-up pulls, one at 50% and one at 75% of their perceived maximum effort, separated by one minute of rest. Once body position was stable, the subjects were given a countdown of “3, 2, 1, pull”. Minimal pre-tension was encouraged to ensure that there was no slack in the subject’s body or IMTP rig before initiation of the pull (defined as the instant when force exceeded a threshold equal to five times the standard deviation of bodyweight [24]). Subjects then performed three maximal IMTPs, with the instruction to pull against the bar as fast and hard as possible whilst synchronously pushing the feet down into the force platform. Each maximal IMTP trial was performed for five seconds and interspersed by two minutes of rest. Trials were repeated if the peak force values varied by >250 N, in line with previous research [21,22,25,26]. 

### 2.3. Data Collection

All CMJs and IMTPs were recorded at 1000 Hz using a Kistler type 9286AA force platform and Bioware 5.11 software (Kistler Instruments Inc., Amherst, NY, USA). For the CMJs and IMTPs, subjects were instructed to stand still for the initial one second of data collection [27,28] to enable the subsequent determination of body weight (vertical force averaged over 1 s). All raw vertical force-time data were subsequently exported as text files and analyzed using a customized Microsoft Excel spreadsheet (version 2016, Microsoft Corp., Redmond, WA, USA). 

### 2.4. Data Analysis

For the CMJ data, the center of mass (COM) velocity was determined by dividing vertical force data (minus body weight) by body mass and then integrating the product using the trapezoid rule. Instantaneous power was calculated by multiplying vertical force and velocity data at each time point and COM displacement was determined by twice integrating vertical force data [28]. The start of the CMJ was identified in line with current recommendations [27]. The braking phase of the CMJ was defined as occurring between the instants of peak negative COM velocity and zero COM velocity. The propulsion phase of the CMJ was deemed to have started when COM velocity exceeded 0.01 m s^−1^ and finished at take-off [11,16,17]. Take-off was identified when vertical force fell below five times the standard deviation of the flight phase force [11,16,17,28].

Braking and propulsion mean and peak force, power, velocity, and displacement were defined as the maximum and mean values attained during the braking and propulsion phases, respectively [11,16,17]. Net impulse was calculated during both the braking and propulsion phases as the area under the net force-time curve (minus body weight) using the trapezoid rule [9]. Jump height was derived from vertical velocity at take-off [28]. Reactive strength index modified (RSI_mod_) was calculated as jump height divided by TTT (i.e., the time between the onset of movement and take-off) [29]. 

The TPA of the three CMJ trials was conducted by modifying individual force-, velocity-, power‑, and displacement-time curves from the onset of movement to the instant of take-off so that they each equaled 500 samples [16,17,20]. This was achieved by changing the time delta between the original samples (e.g., original number of samples/500) and subsequently re-sampling the data [11,16,17,20]. This resulted in an average sample frequency of 688 ± 87 Hz and 720 ± 120 Hz for the high and low DSI groups’ data, respectively, and allowed the averaged curve of each variable to be expressed over a percentage of normalized time (e.g., 0–100% of TTT). 

For the IMTP data, the maximum force recorded from the force-time curve during each five-second trial was reported as the peak force. All kinetic data (CMJ and IMTP) were normalized by dividing them by body mass to enable group comparisons. The DSI was calculated by dividing CMJ propulsion peak force by IMTP peak force.

### 2.5. Statistical Analysis

For each gross measure and the TPA, the mean output of the three CMJ trials was taken forward for statistical analysis. All data satisfied parametric assumptions, except propulsion COM displacement for the low DSI group. Mean differences in each parametric variable derived for high and low DSI groups were, therefore, compared using independent *t*-tests, whereas propulsion COM displacement was compared between groups via the Mann-Whitney U test. A two-way random-effects model ICC was used to determine the relative between-trial reliability of each variable and interpreted according to previous work where a value of ≥0.80 is considered highly reliable [30]. Independent t-tests, the Mann-Whitney U test, and ICCs were performed using SPSS software (version 20; SPSS Inc., Chicago, IL, USA) with the alpha level set at *p* ≤ 0.05. Absolute between-trial variability of each gross variable was calculated using the coefficient of variation (calculated in this study as the standard deviation divided by the mean) expressed as a percentage (%CV). A CV of ≤10% was considered to be reflective of acceptable variability, in line with previous recommendations [31]. Effect size (ES) calculations (Cohen’s *d*) were calculated to provide a measure of the magnitude of the differences between groups for each variable and interpreted in line with previous recommendations which defined values as trivial (≤0.19), small (0.20–0.59), moderate (0.60–1.19), large (1.20–1.99), and very large (2.0–4.0) [32]. Likely group differences in force-, velocity-, power-, and displacement-time curves were determined by plotting the time normalized average curves for each group along with the corresponding upper and lower 95% confidence intervals to create upper and lower control limits and identifying non-overlapping areas [11,17,33]. 

## 3. Results

The mean DSI for the entire subject group (*n* = 53) was 0.73 ± 0.19, whereas the mean DSI for the low (*n* = 20) and high DSI (*n* = 20) groups was 0.55 ± 0.10 and 0.92 ± 0.11 (*p* < 0.001, *d* = 3.54), respectively. DSI demonstrated high reliability (ICC = 0.937) and acceptable variability (%CV = 6.1). The mean IMTP peak force for the low and high DSI groups was 46.7 ± 15.0 N kg^−1^ and 31.1 ± 6.6 N kg^−1^ (*p* < 0.001, *d* = 1.35), respectively. IMTP peak force also demonstrated high reliability (ICC = 0.952) and acceptable variability (%CV = 4.7).

All CMJ variables demonstrated high reliability and acceptable variability (Table 2). The low DSI group demonstrated significantly greater COM displacement, power, velocity, and impulse in the braking phase, in addition to significantly greater phase time, COM displacement, velocity, and impulse in the propulsion phase (Table 2). There were no significant differences in any other CMJ variables between low and high DSI groups (Table 2).

**Figure 1:**
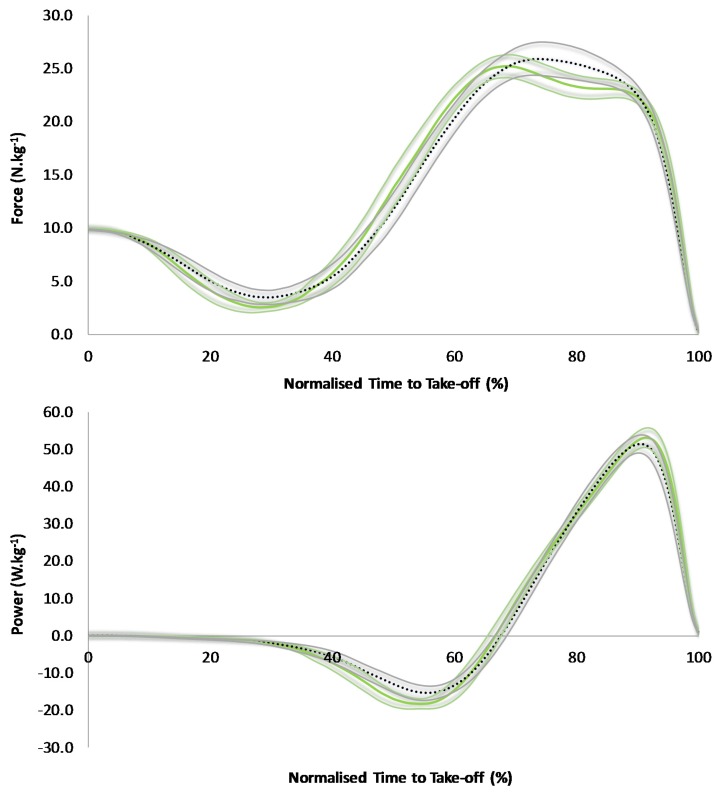
A comparison of countermovement jump force-time (**top**) and power-time (**bottom**) curves between the low (green solid line) and high (black dotted line) DSI groups, along with shaded 95% confidence intervals.

**Figure 2:**
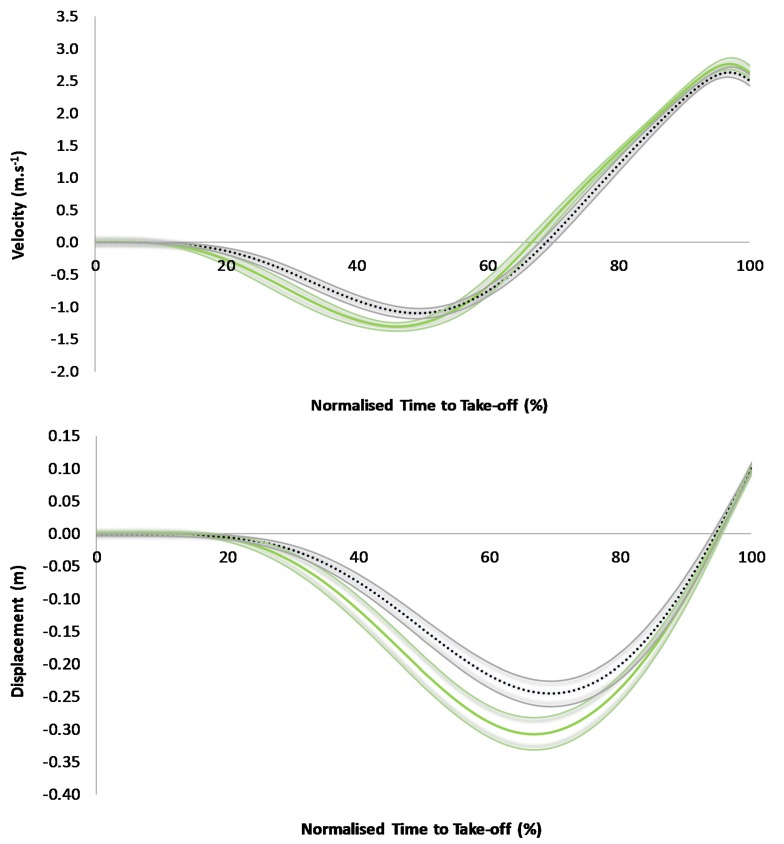
A comparison of countermovement jump velocity-time (**top**) and displacement-time (**bottom**) curves between the low (green solid line) and high (black dotted line) DSI groups, along with shaded 95% confidence intervals.

The TPA of CMJ revealed no areas of no overlap for force and power between low and high DSI groups (Figure 1). The low DSI group demonstrated a greater negative velocity between 25% and 49% of normalized TTT, however, which corresponded to most of the unweighting phase for both groups and the onset of braking phase for the low DSI group (Figure 2). The low DSI group then demonstrated a greater positive velocity between 70% and 78% of normalized TTT which corresponded to the early portion of the propulsion phase (Figure 2). Additionally, the low DSI group demonstrated greater negative COM displacement between 35% and 75% of normalized TTT which corresponded to the late unweighting phase through to the early propulsion phase (Figure 2). 

## 4. Discussion

The purpose of this study was to quantitatively describe the influence of DSI on CMJ force-, power-, velocity-, and displacement-time curves by comparing these curves, using the TPA approach, between athletes who achieved differing (i.e., high versus low) DSI values. To the authors’ knowledge, this is the first study to conduct a TPA of CMJ performances by subjects who attained a high versus a low DSI. The variable which demonstrated the largest difference between the high and low DSI groups was braking impulse, with a larger value noted for the low DSI group (Table 2). As braking impulse equals unweighting impulse [34,35], the low DSI group would have demonstrated a greater unweighting impulse, thus requiring them to generate a greater braking impulse. Evidence of this can be seen in the velocity-time curve analyses, whereby the low DSI group showed greater negative velocity (i.e., greater impulse equals greater velocity of a given mass) throughout most of the unweighting phase (Figure 2). Owing to the COM travelling a greater velocity but over a similar duration, greater displacement was noted in the late unweighting phase (Figure 2). The greater braking impulse (slightly lower force applied over a slightly longer duration) also led to greater negative velocity during the early braking phase and greater COM displacement throughout the entire braking phase (Figure 2). Consequently, braking peak power was significantly greater for the low DSI group (Table 2), however, this was not reflected in the power-time curve analyses whereby 95% CIs are also considered (Figure 1). Nevertheless, the results suggest that the increase in braking work (similar force but more displacement) outweighed the increase in braking time, resulting in a subtly higher braking power for the low DSI group. 

In the propulsion phase, the low DSI group produced a greater net impulse that was characterized by a slightly lower peak force (with this variable forming part of the DSI calculation) and a longer phase time (Table 2). Of course, these propulsion phase characteristics will have been influenced by the preceding braking phase characteristics, particularly in terms of countermovement displacement, as starting from a lower COM position at the onset of the propulsion phase generally leads to a reduction in propulsion peak force and an increase in propulsion phase time [9,10], like what has been shown here. Consequently, a greater peak velocity and COM displacement during the propulsion phase, with respect to gross values, was attained by the low DSI group, but differences were only noted during the early part of the propulsion phase with respect to the TPA (Figure 2). This strategy led to just a moderately greater jump height (~3.5 cm) for the low DSI group. Overall, except for phase times and peak forces, all other variables showed larger between-group differences in the braking phase versus the propulsion phase based on the effect sizes (Table 2). Considering these results, it seems that braking phase characteristics best distinguish between those who attain a high versus a low DSI score. Consequently, the original hypotheses of the study were partially accepted in that the high DSI group did produce lower velocity and countermovement displacements, both with respect to gross values and the TPA, but they did not produce higher force and power, with the latter being higher for the low DSI group in the braking phase. 

The opening paragraphs of this discussion focus on describing how propulsion peak force during the CMJ was attained by the high and low DSI groups by considering the kinetic and kinematic strategy employed by each group during both the braking and propulsion phases. Before discussing the efficacy of the DSI metric, in relation to its intended purpose of describing whether athletes should focus on ballistic or maximal strength training and in light of the present results, first, a brief discussion about the IMTP peak force values attained by the low and high DSI groups is warranted, as these should also be considered before providing training recommendations. In this study, the low DSI group were largely stronger (higher IMTP peak force) than the high DSI group, possibly due to the latter having fewer years of resistance training experience (Table 1). Thus, the low DSI group achieved a lower DSI than the high DSI group due to a combination of them being stronger and producing a slightly lower propulsion peak force in the CMJ. In other words, the low DSI group had a much larger capacity to produce force in the CMJ, but they actually produced marginally less force than the high DSI group. To further facilitate the interpretation of the IMTP peak force values presented here, the mean IMTP peak force (relative to body mass) for the low DSI group was close to values attained by a “stronger” group of collegiate athletes tested by Beattie et al. [36]. Additionally, mean IMTP peak force (relative to body mass) for the high DSI group was comparable to values attained by groups of collegiate athletes defined as “weaker” by both Thomas et al. [37] and Beattie et al. [36]. It might be deemed, therefore, that the low and high DSI groups were relatively strong and weak, respectively. Intuitively then, maximal strength training would seem more obviously suited to the high DSI group [18] based on IMTP peak force scores alone, but the impact of maximal strength training and ballistic training on both IMTP peak force and CMJ propulsion peak force should be considered if the aim is to alter DSI. 

The first ballistic training intervention study to present a comprehensive collection and analyses of performance data revealed that when ballistic training alone was performed (12 weeks), relative strength remained unchanged (one repetition maximum (1RM) back squat/body mass) and only a slight but non-significant increase in CMJ relative propulsion peak force was reported [19]. The non-significant increase in CMJ relative to propulsion peak force was likely due to subjects altering their CMJ strategy in the way of increased countermovement displacement [19]. This group would have, therefore, likely observed only a slight increase in DSI (similar strength but slightly greater CMJ force production). A later study [18], which compared maximal strength training to ballistic training, showed that the strength training group increased back squat 1RM, isometric squat peak force, and CMJ relative propulsion peak force, whereas the ballistic training group only increased CMJ relative propulsion peak force, albeit to a larger extent. Both strength and ballistic training groups maintained a similar countermovement displacement post-training (10 weeks), but the ballistic training group also demonstrated a reduced CMJ TTT, hence they were able to produce a more force-dominant propulsion impulse post-intervention [18]. Given the moderate (relative force) to large (absolute force) relationships between isometric squat peak force and IMTP peak force reported in previous work [38], it can be reasonably deduced that the ballistic training group would have likely increased their DSI score (isometric peak force stayed the same but CMJ propulsion peak force increased, thus bringing these values closer together), whereas the strength training group would have decreased their DSI score slightly (increased isometric squat peak force by ~5.1 N·kg^−1^ and CMJ propulsion peak force by ~3.0 N kg^−1^). 

When stronger (relative back squat 1RM of 1.97 kg·kg^−1^) and weaker (relative back squat 1RM of 1.32 kg·kg^−1^) subjects where compared following the completion of a ballistic training program (10 weeks), it was revealed that both groups significantly increased CMJ propulsion peak force, but the stronger group showed marginally larger improvements [7]. This increase in peak propulsion force was accompanied by a reduction in TTT for both groups and similar and slight reduction in countermovement displacement (based on visual inspection of figures by the present authors) for the weaker and stronger groups, respectively [7]. Interestingly though, back squat 1RM reduced for the strong group (ES = 0.93) but increased slightly for the weaker group (ES not reported) [7]. Therefore, DSI would have likely increased for the stronger group but may have remained similar for the weaker group. The above-mentioned results somewhat support the efficacy of prescribing ballistic training to athletes with low DSI scores and strength training to athletes with high DSI scores, but the consideration of maximal strength level is also warranted and a combined maximal strength and ballistic training approach may be more effective for improving overall athleticism. Ballistic training also helps to improve the coupling of braking-propulsion phase kinetics [18] which may help those with a low DSI who, in line with the present results (Table 2), may put a lot more into the braking phase but gain little advantage in the proceeding propulsion phase [39]. Future research avenues should include exploration of the impact of maximal strength, ballistic, and concurrent (maximal strength and ballistic) training on DSI and CMJ phase characteristics. The present study could also be replicated with elite athletes to confirm whether high and low DSI scorers among this population demonstrate similar CMJ phase characteristics to the subjects tested here.

## 5. Conclusions

The low DSI group were stronger but performed the CMJ with a braking phase characterized by a larger net impulse, leading to greater braking velocity and countermovement displacement. This strategy led to the attainment of a similar propulsion peak force to the weaker high DSI group. Based on the present results, and on the results of previous training studies [7,18,19], it seems efficacious for low DSI scorers to prioritize ballistic training but maximal strength should at least be maintained for this group. High DSI scorers would likely benefit most from a maximal strength training program to increase isometric peak force capacity followed by ballistic training to increase peak force production in the CMJ. Irrespective of a given DSI score attained by an athlete, it would be prudent for both their lower body strength and CMJ strategy to also be considered to more accurately inform their precise short-term training needs.

## Figures and Tables

**Table 1:** Physical characteristics of all subjects and each group (mean ± standard deviation).

	All Subjects (*n* = 53)			Low DSI Group (*n* = 20)			High DSI Group (*n* = 20)		
Age (years)	23.1	±	4.1	24.4	±	4.3	20.4	±	1.1
Height (m)	181.5	±	6.1	181.5	±	6.1	181.6	±	6.8
Body Mass (kg)	78.3	±	9.6	80.0	±	10.8	76.6	±	8.0
RT Experience (years)	3.4	±	2.9	4.5	±	3.7	2.4	±	1.4

DSI = dynamic strength index; RT = resistance training.

**Table 2:** A group comparison of gross linear kinetic and kinematic countermovement jump variables.

Jump Variables	Low DSI		High DSI		*p*	*d*	ICC	%CV
	Mean	SD	Mean	SD				
Jump Height (cm)	35.9	6.2	32.4	5.0	0.062	0.62	0.937	3.7
Time to Take-off (s)	0.737	0.102	0.679	0.081	0.055	0.63	0.862	4.4
RSI_mod_ (ratio)	0.49	0.08	0.46	0.08	0.275	0.35	0.819	6.5
Braking Phase Time (s)	0.151	0.021	0.138	0.027	0.110	0.52	0.860	6.0
Propulsion Phase Time (s)	0.240	0.034	0.213	0.030	0.011	0.85	0.927	3.7
Braking COM Displacement (cm)	31.0	5.8	25.3	5.3	0.002	1.02	0.919	5.5
Propulsion COM Displacement (cm)	41.0	6.8	35.3	5.3	0.005	0.93	0.943	3.7
Braking Peak Force (N·kg^−1^)	25.5	2.5	25.4	3.1	0.926	0.03	0.815	4.2
Propulsion Peak Force (N·kg^−1^)	25.9	2.2	27.0	3.3	0.202	0.41	0.890	3.2
Braking Peak Power (W·kg^−1^)	20.9	4.8	17.4	3.7	0.014	0.81	0.845	8.5
Propulsion Peak Power (W·kg^−1^)	54.4	5.8	53.0	5.7	0.430	0.25	0.928	3.7
Braking Peak Velocity (m·s^−1^)	1.37	0.20	1.18	0.16	0.002	1.08	0.880	4.9
Propulsion Peak Velocity (m·s^−1^)	2.78	0.21	2.65	0.18	0.044	0.66	0.944	1.5
Braking Impulse (N·kg^−1^·s)	1.38	0.21	1.18	0.16	0.001	1.10	0.883	5.0
Propulsion Impulse (N·kg^−1^·s)	2.60	0.24	2.43	0.20	0.027	0.70	0.936	2.0

SD = Standard Deviation; ICC = Intraclass Correlation Coefficient; %CV = Percentage Coefficient of Variation; RSI_mod_ = Reactive Strength Index Modified; COM = Center of Mass.
